# The hand that ‘sees’ to grasp

**DOI:** 10.7554/eLife.18887

**Published:** 2016-07-29

**Authors:** Kenneth F Valyear

**Affiliations:** School of Psychology, Bangor University, Bangor, United Kingdomkfvalyear@gmail.com

**Keywords:** parietal cortex, premotor cortex, motor cortex, sensorimotor transformation, parallel recording, hand grasping, Rhesus macaque

## Abstract

New findings advance our understanding of how vision is used to guide the hand during object grasping.

**Related research article** Schaffelhofer S, Scherberger H. 2016. Object vision to hand action in macaque parietal, premotor, and motor cortices. *eLife* **5**:e15278. doi: 10.7554/eLife.15278

**Image** A specialized glove measures the hand movements made by monkeys as they grasp an object

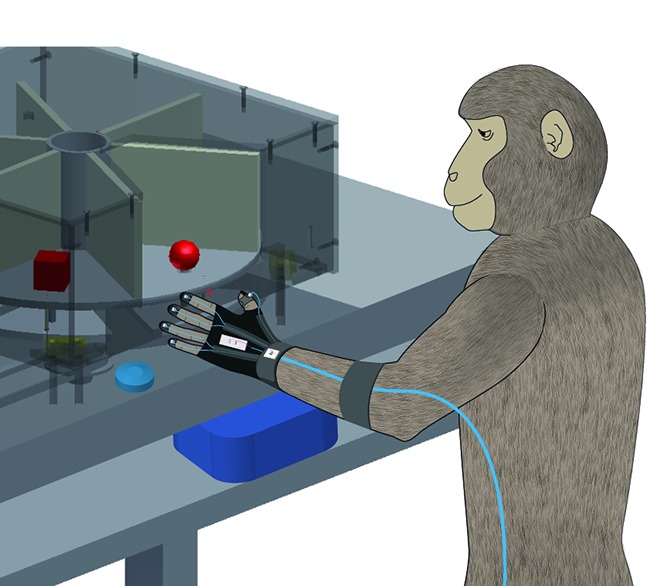

How does the hand know what the eyes see? When we reach to grasp an object, our hand shapes to match the object's size, shape, and orientation. How does the brain translate visual information into motor commands that control the hand?

Now, in eLife, thanks to the work of Stefan Schaffelhofer and Hansjörg Scherberger of the German Primate Center in Göttingen, our understanding of this fundamental process has been significantly advanced (Schaffelhofer and Scherberger, 2016). We can think about the information that is represented in the activity of cells as a ‘code’. For the first time, cells that code for the visual properties of objects are distinguished from those that code for how the hand is moved to grasp.

The approach used by Schaffelhofer and Scherberger records the hand movements and the activity of brain cells in monkeys while they view and grasp objects of different shapes and sizes. Recordings are taken from three brain areas known to be important for grasping – the anterior intraparietal (AIP) area, the ventral premotor area F5 and the primary motor hand area M1 (Figure 1).

**Figure 1.**
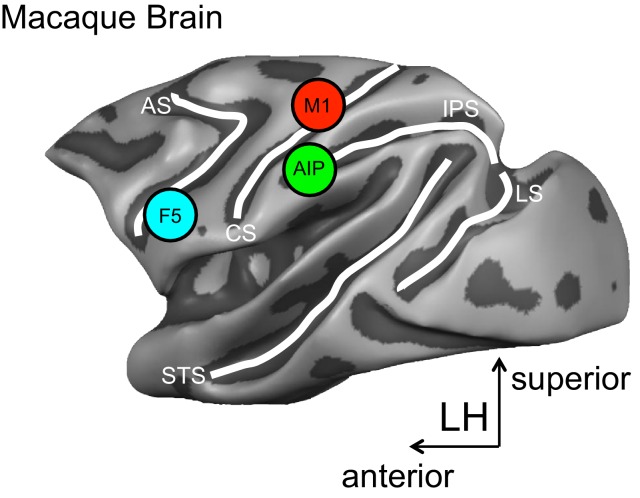
A schematic representation of the brain areas implicated in the transformation of visual-to-motor information during grasping. The cortical surface of the macaque monkey is shown. The cortical surface is defined at the gray-white matter boundary and has been partially inflated to reveal regions within the sulci (the grooves on the brain’s surface) while preserving a sense of curvature. AIP = anterior intraparietal area, F5 = ventral premotor area, M1 = primary motor hand area. White lines indicate sulci. IPS = intraparietal sulcus, STS = superior temporal sulcus, CS = central sulcus, AS = arcuate sulcus, LS = lunate sulcus. LH = left hemisphere. The monkey MRI data on which the reconstruction is based was provided by Stefan Everling.

The set of objects used in the study elicits a wide range of different hand postures. Critically, some objects look different but are grasped similarly, while others look identical but are grasped in different ways. This approach allows for cells that represent visual object properties (visual-object encoding) to be distinguished from those that represent how the hand is moved during grasping (motor-grasp encoding).

The results reveal predominately visual-object encoding in area AIP and motor-grasp encoding in areas F5 and M1. The cells in area AIP respond strongly during object viewing, and these responses clearly reflect object shape. Conversely, area F5 responds only weakly during the viewing period, and its activity strongly reflects hand movements during grasping.

It is particularly informative that when the objects are visually distinct but are grasped similarly, each object initially causes a distinct response in area AIP during the viewing period. These responses become more similar over time and before the start of a movement. This is consistent with a change from a visual-object to a motor-grasp encoding scheme. Area AIP also responds differently to visually identical objects that are grasped differently, which is also consistent with a motor-grasp encoding scheme.

Despite these aspects of their results, Schaffelhofer (who is also at Rockefeller University) and Scherberger (who is also at the University of Göttingen) maintain that altogether their data more strongly support a visual-object encoding account of AIP activity. They suggest that area AIP represents the visual features of objects that are relevant for grasping.

According to this account, over time AIP activity reflects a narrowing of action possibilities, honing in on the object features that will be essential for the upcoming grasp. This explains why responses to different objects that are grasped similarly become increasingly similar during planning, and why grasping the same object in different ways elicits distinct responses.

The findings also reveal that areas AIP and F5 briefly show common encoding when viewing objects, suggesting that these areas share information during this time. Speculatively, feedback from F5 may help to narrow the range of possible hand actions (specified visually in area AIP) to a single set of grasp points on the target object.

The results of Schaffelhofer and Scherberger also provide compelling evidence for the role of area F5 in driving the activity of the primary motor area M1. Response encoding during grasping becomes remarkably similar between areas F5 and M1, and F5 responses show earlier onsets. These data complement and extend previous results (Umilta et al., 2007; Spinks et al., 2008).

Altogether the new findings suggest the following model. Area AIP represents visual information about the features of objects. Together with area F5, area AIP then ‘flags’ those features that are most relevant for the intended actions, and the two areas collaborate to transform this information into the sensory and motor parameters that control the hand during grasping. Finally, area F5 signals this information to area M1, and ultimately the information reaches the spinal cord for controlling the hand and finger muscles.

This model can now be further tested. For example, the model predicts that area AIP will respond robustly and variably to objects that can be grasped in many different ways. The model also suggests that the more difficult it is to visually identify the parts of an object that will permit a stable grasp, the more rigorously AIP will respond.

It would also be interesting to see how the interplay between areas AIP and F5 unfolds when grasping the same object for different purposes (Marteniuk et al., 1987; Ansuini et al., 2006). Does the activity in area AIP first represent all the visual features of the object, as the current results of Schaffelhofer and Scherberger suggest? Or are the responses in area AIP adjusted to reflect those features of the object that are most relevant to the specific action that is intended?
